# Effects of Silver Nanoparticle on the Developing Liver of Rat Pups after Maternal Exposure

**Published:** 2017

**Authors:** Mahnoosh Fatemi, Jamal Moshtaghian, Kamran Ghaedi, Narges Jafari dinani, Gholamali Naderi

**Affiliations:** a *Department of Biology, Falavarjan Branch, Islamic Azad University, Isfahan, Iran.*; b *Biology Department, School of Sciences, University of Isfahan, Isfahan, Iran.*; c *Department of Cellular Biotechnology at Cell Science Research Center,Royan Institute for Biotechnology, ACECR, Isfahan, Iran.*; d *Cardiovascular Research Center, Cardiovascular Research Institute, Isfahan University of Medical Sciences, Isfahan, Iran. *; e *Isfahan Cardiac Rehabilitation Center, Cardiovascular Research Institute, Isfahan University of Medical Sciences, Isfahan, Iran.*

**Keywords:** Apoptosis, Developmental hepatotoxicity, Liver, Oxidative stress, Rat

## Abstract

The extensive application of silver nanoparticles (AgNPs) has been increased due to their antimicrobial properties, however numerous concerns has been arisen about their toxicity potential. Since nanoparticles can cross through the placenta and accumulate in the embryonic organs especially liver, in this study, developmental hepatotoxicity of AgNPs was investigated.

Pregnant rats were divided into two groups, vehicle control group and treated group. Treated group received AgNPs (25 mg/Kg) through intra-gastric gavage in a period of gestational days 1-19. Pups were sacrificed after the birth and their livers collected. Histopathology, ICP- mass spectrometry, Spectrophotometric assay, and ELISA were employed to evaluate AgNPs toxicity in the liver of pups.

Glutathione peroxidase (GPX) activity and glutathione (GSH) level were significantly decreased and malondialdehyde (MDA) and caspase 9 levels were significantly increased, although there was no significant change in caspase 8 content in the liver of offspring. Fatty degeneration and congested dilated sinusoids were also observed in histo-pathological examination.

These results suggest that maternal oral exposure to AgNPs may induce oxidative stress and apoptosis in the liver of their offspring. Further investigations are required to clarify molecular events behind this happening.

## Introduction

In the recent years, silver nanoparticles have been widely used due to their antimicrobial properties even in medicine and food industry (1).

The widespread use of these particles increases their potential toxicity. Some medical and biological reports have proved that nanoparticles transport into the body via inhalation, ingestion, and dermal route as well as produce adverse effects (2).

Previous studies on experimental animals and *in vitro* have reported that toxicity of silver nanoparticles is related to oxidative stress induction (3, 4), apoptosis (5, 6) and induction of lipid peroxidation (7).

Oxidative stress is an imbalance between the reactive oxygen species (ROS) generation and antioxidant defense that can lead to lipid peroxidation and even can trigger apoptosis or necrosis (8, 9). Unfortunately, few studies focused on the effects of prenatal exposure to nanoparticles, especially AgNPs on the developing fetus. It is well known that tissues and organs are more susceptible to toxic factors in the prenatal stage than mature age (10) and also recent studies have shown that nanoparticles can penetrate the placental barrier and accumulate in the embryonic tissues (11, 12 and 13). Therefore, the aim of this study was evaluation of the hazardous maternal exposure to AgNPs in the liver of the rat offspring.

## Experimental

*Materials*

Silver, dispersion nanoparticle (20 ± 4 nm) in colloidal form (0.02 mg/mL) containing sodium citrate as an stabilizer, based on the datasheet which was provided by Sigma Aldrich, Company (Prod. No.730793). ELISA kits for assessment of the caspase 8 and caspase 9 were purchased from Glory science Company. Meanwhile, all chemicals used for the spectrophotometric and histopathological analysis were purchased from Sigma Aldrich unless stated otherwise.

 Treatment procedure: All animal experiments were conducted under a protocol approved by the Institutional Review Board (IRB) of the Islamic Azad University Falavarjan Branch (No: 301/28185).

Adult male and female Wistar rats (180 ± 20 g) were purchased from the Animal Center of Isfahan University and acclimated for three weeks prior to the initiation of the study. Rats were housed under controlled environmental conditions with a temperature of 23 ± 2 ºC, a 12:12 h light/dark cycle and relative humidity of 55 ± 5%. Food and water for rats were provided *ad libitum*. Time-mated pregnant rats were randomly divided into two groups, a control group and AgNPs treated group. Thirty pregnant rats from each of the control and treated groups were gavaged with deionized water and AgNPs (dose of 25 mg/Kg, body wt. per day) in a period of gestational days 1-19, respectively. Gestation begins with the sign of vaginal plug as evidence of copulation or gestation day 0. The size and agglomeration state of AgNPsafter preparation of concentration of 25 mg/Kg were determined using Transmission Electron Microscopy (LEO912-AB/Zeiss Germany). No changes were observed in agglomeration state and particle size compared with the manufacturer´s information. After delivery, one male pup (per each litter with 6-8 pups) was randomly chosen from each group. The pups (16 pups per group) were weighed, sacrificed and their livers were dissected out. The livers were weighed and the ratio of liver weight to body weight was calculated. Then, the livers were quickly frozen at -80 ºC. 

*Histopathological analysis:*

Animals livers were fixed in neutral buffered formalin (10%), dehydrated with ethanol and cleaned with xylene. The samples were embedded in paraffin and were sliced into 5 µM sections (8 slices from each liver). The paraffin sections were stained with hematoxylin and eosin and were studied under a light microscope.

*Silver content analysis:*

The animals’ livers were digested with nitric acid (1 M) and perchloric acid (1 M) (at a ratio of 4:1 v/v) for 48 h and were incubated at 120 ºC (to remove the remaining acids). Each sample was diluted with distilled water (1 mL) and used for silver total mass measurement by Inductively Coupled Plasma Mass Spectrometry (ICP-MS) (14).

*Determination of oxidative stress and apoptosis biomarkers:*

The livers of the pups in each of the control and treated groups were weighed and homogenized (1:10 w/v) in potassium phosphate buffer (0.1 M, pH = 7.4, 4 ºC). The homogenates were centrifuged at 3000 rpm for 10 min at 4 ºC, the supernatants were used for assessment of oxidative stress and apoptosis.

The levels of glutathione in the livers were measured by spectrophotometric assay. One mL of the supernatant was mixed with tri-chloroacetic acid (5% w/v) and then centrifuged to remove the proteins. One hundred µL of this mixture was added to a volume of 0.4 mL of double distilled water, 0.5 mL 5,5›- dithiobis- (2- nitrobenzoic acid and 2 mL of phosphate buffer (0.5 M, pH = 8.4). Subsequently, the samples were vortexed and the absorbance at 412 nm was recorded within 15 min (15).

**Figure 1:**
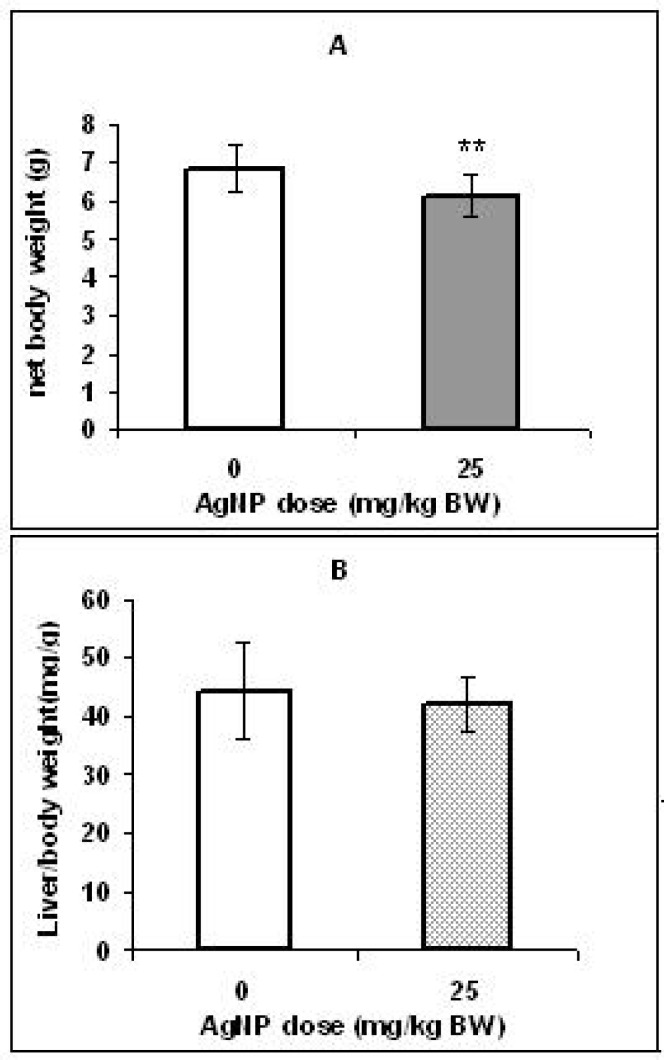
Effect of AgNPs on net weight (A) and liver/body weight ratio (B) of offspring rats. Bars marked with a star show significant different from control group (** *p *< 0.01). Values are means ± SD. N = 16.

**Figure 2:**
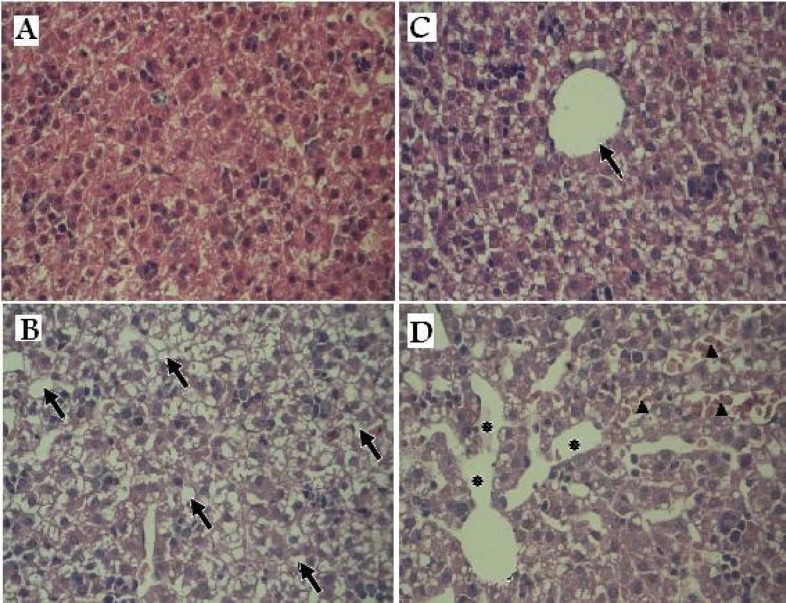
Histopathology of liver tissues in offspring rats exposed prenatally to vehicle or AgNPs. (A & C) control group: Normal hepatic lobule and hepatocytes surrounding a central vein (arrow). (B & D) treated group: Arrows indicate vacuolated appearance (B).Asterisks denote the massive destination in the sinusoid space and arrows indicate congested hepatic sinusoids containing red blood cells. (×100

**Figure 3:**
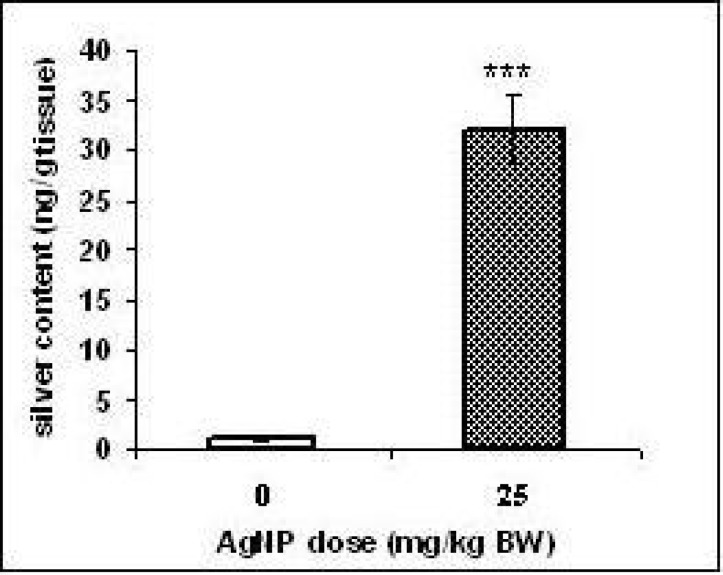
Silver contents in rat livers after developmental AgNPs exposure. Values represent means ± SD. N = 8. ***p< 0.001(significantly different from control group

**Figure 4:**
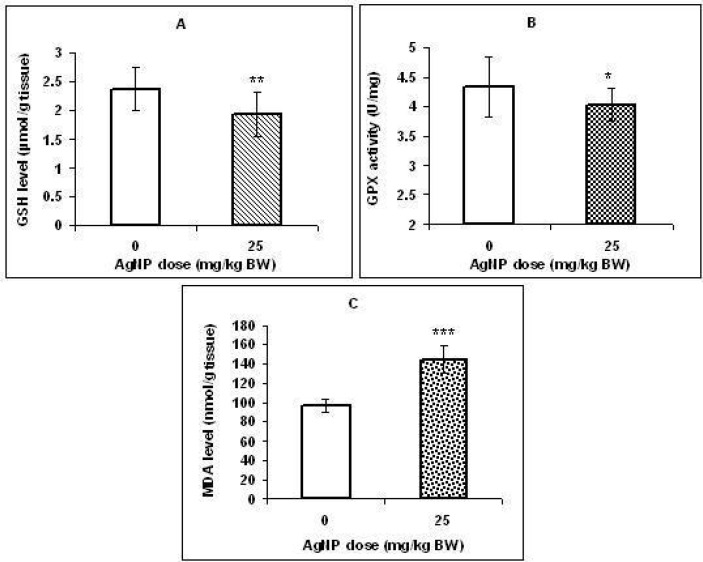
Effect of AgNPs on the GSH level (A), GPX activity (B), and MDA level (C) in the rat offspring livers that prenatally exposed to AgNPs. Values represent means ± SD. N = 16. *p < 0.05, ** *p *< 0.01, ***p < 0.001 (significantly different from control group

**Figure 5:**
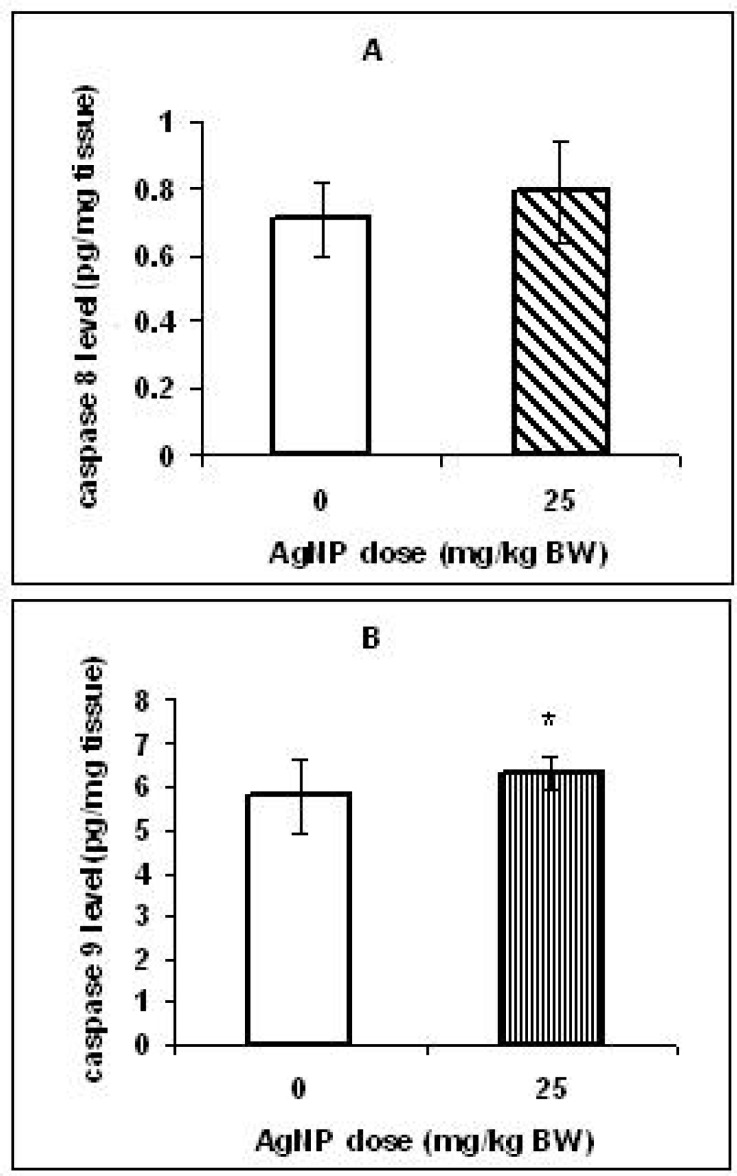
Effect of AgNPs on the caspase 8 (A) and caspase 9 (B) levels in the offspring livers that prenatally exposed to AgNPs. Values represent means ± s.d., n = 16. * *p *< 0.5

To measure glutathione peroxidase (GPX) activity, 1 mL of 0.4 M phosphate buffer (pH = 7.0) containing 1 mL reduced glutathione (8 mM), EDTA (0.4 mM) and 1 mL of NaN_3_ (5 mM) was mixed with 0.2 mL of supernatant and then was incubated at 37 ºC for 10 min. Thereafter, 1 mL of H_2_O_2_ (8 mM) and the mixture for 10 min was incubated at 37 ºC. The excess amount of GSH (as described above) was quantified by the method of DTNB. The amount of enzyme required to oxidize 1 nmol GSH/per minute is equal to one unit of enzyme activity (16). Malondialdehyde was measured by using the thiobarbituric acid assay. Briefly, 1 mL of 50% tri-chloroacetic acid in 1 mL of thiobarbituric acid (26 mM) and HCl (0.1 M) was added to 200 µL of supernatant. The samples were vortexed and maintained at 95 ºC for 20 min. After centrifugation (at 1000 ×g) for 10 min, supernatants were collected and the absorbance was recorded at 532 nm (16). 

Measurement of caspase 8 and caspase 9 contents in the livers was performed based on the instructions provided by the manufacturer of the ELISA kits (Glory Science Co., Ltd in China). Briefly, the liver homogenates after two freeze- thaw cycles (to break the cell membranes) were centrifuged at 5000 × *g* for 5 min. Forty µL of supernatant, 10 µL of visfatin- antibody and 50 µL of streptavidin- HRP were added to test wells and a volume of 50 µL of streptavidin- HRP and 50 µL of standard were added to standard wells. The plates were incubated at 37 ºC for 60 min. After washing, 50 µL of chromogen solution A, then 50 µL of chromogen solution B was added to each well and incubated at 37 ºC for 10 min away from light. Absorbance of the samples was measured and calculated according to standard curves, after addition of 50 µL of stop solution (to stop the reaction).

*Statistical analysis:*

The data for each group were expressed as mean ± standard deviation. The independent *t*-test was used for data analysis and all analyses were performed using the Statistical Package for the Social Sciences (SPSS) version 16.0 software. The statistical significance of all data was set at *p < 0.05*, *p < 0.01* and *p < 0.001*. Excel 2003 software was used for producing diagrams.

## Results

*The ratio of liver weight to body weight:*

Figure 1A shows that body weight in the pups of the control group was significantly higher (*p < 0.01*) than the pups in the treated group. However, no significant differences were found in the coefficients of the liver to body weight of two groups (Figure 1B). 

*Histological alterations:*

Figure 2 shows histopathological findings of offspring’s livers of the control and treated groups. At the microscopic level, the liver of the treated group pups presented the histopathological alterations as congested dilated blood sinusoids and vacuolated appearance of the liver tissue. These results suggest that AgNPs accumulation in the liver might lead to liver damage.

*Silver content in the liver:*

Silver levels were higher in the liver of the rat’s offspring of the exposed group (*p < 0.001*) than the unexposed group (Figure 3). These results indicated that AgNPs traversed into the liver.

*Effect of AgNPs on oxidative stress and apoptosis biomarkers:*

As shown in figure 4 A & B, a significant decrease (*p < 0.01*) in the concentration of GSH and GPX activity (*p < 0.05*) was observed in the liver of treated pups compared with those in the control group. Inaddition, MDA level in the liver was significantly higher in pups exposed to AgNPs (*p < 0.001*; Figure 4C).

To investigate the relation between toxicity caused by AgNPs and apoptosis induction, the caspase 8 & 9 concentrations were measured in the livers. Although, no marked difference was observed in the caspase 8 level between the two groups (Figure 5A), there was a significant increase (*p < 0.05*) in caspase 9 level in the livers of the treated group offspring compared with those in the control group (Figure 5B).

## Discussion

The oral ingestion is a potential route of the human exposure to silver nanoparticles (17). Furthermore, recent studies have revealed that silver nanoparticles are able to penetrate across the placental barrier into the fetus organs after oral administration to pregnant females (13, 18 and 19). Therefore, in this study we assessed the effects of maternal exposure to AgNPs via intra-gastric route (on gestation) on the liver of the rat’s offspring. Our findings indicated that while the liver/body weight ratio in pups in the AgNPs treated group did not change significantly, their body weight decreased significantly compare to the control group.

In several studies, the decrease in the body weight and neonatal growth exhibited after prenatal exposure to various nanoparticles (9, 20 and 22). In the present work, reduced body weight may be associated with fetal toxicity (directly) by placental translocation of nanosilver.

Fetal liver is a potential target site to receive the maternal blood from the placenta (23). Therefore, we evaluated probability of the silver penetration to the offspring’s liver by ICP-MS. The results showed that the content of the silver was raised significantly (*p < 0.001*) in the pups liver of treated group compared to those of the control group. However, it was not investigated whether the silver reached the livers as AgNPs. It is believed that Ag^+^ ions could be released from silver nanoparticles in the aqueous media and into the stomach fluid (15, 24, 25 and 26). The microscopic study of the H & E staining slices showed widely dilated blood vessels, congested hepatic sinusoids containing red blood cells and vacuolated appearance in regions of liver tissues of the treated group offspring that might be due to lipid peroxidation. These changes may confirm that AgNPs lead to liver damage.

Melnik *et al.* (2013) administered [^110m^ Ag]-labeled AgNPs to pregnant rats (20^th^ day of gestation) at a dose of 1.69-2.21 mg/Kg. They observed ^110m^Ag accumulation in rat fetuses 24 h following labeling (13).

Lee *et al*. (2012) also reported that silver nanoparticle (7.9 ± 0.95 nm) penetrates across the placental barrier after oral administration to pregnant rats. These authors observed an increase of 7.9 fold in the silver level in the liver of the treated group pups compared to control group pups (19).

There is evidence that histological changes in liver tissue (in the adult animals) following silver nanoparticles exposure may be associated with oxidative stress (27, 28). Unfortunately, data about developmental hepatotoxicity of AgNPs is lacking. Therefore, since organs in the embryonic stage are highly vulnerable to oxidative stress due to high metabolic rate (29, 30) and it is known that oxidative stress is a main mechanism of silver nanoparticle toxicity (31, 32), in this study, several markers of oxidative stress were measured in the liver tissues. We observed a significant decrease in GSH level and GPX activity as common antioxidants in hepatocytes and a significant increase in MDA level (lipid peroxidation marker) in the liver of the treated group offspring. Our results suggest that the ability of antioxidant defense was probably depressed in the liver tissues which in turn caused lipid peroxidation.

Some *in-vitro* studies have reported ROS generation, decrease in GSH, and lipid peroxidation in many cell lines, including BRL3A cell lines (33), Hep G2 human hepatoma cells (34) and LO2 cell lines (35) following exposure to AgNPs. In line with *in-vitro* findings, Choi *et al*. (2010) evaluated effects of AgNPs administration on the liver of zebra fish. These researchers observed an increase in MDA level and a decrease in total GSH content and gene expression of GPX and catalase (CAT) and suggested that oxidative stress induced in the liver tissues (28). 

Recently, Wu *et al*. (2013) also observed a dose related decrease in the activity of superoxide dismutase (SOD), CAT, GPX level, and an increase in the concentration of MDA after 14 days of exposure of adult Medaka with AgNPs (29.9 nm) at doses of 0.05, 0.1, 0.25 and 0.5 mg/L (27).

The silver nanoparticles are also known to induce DNA damage and apoptosis through oxidative stress-related mechanisms and lipid peroxidation (36, 37, 38, 39). Apoptosis is an important process in embryonic development of organs and structures from invertebrates to mammals. Abnormal apoptosis can lead to developmental injury (40) and a wide variety of diseases, including liver diseases (41). Extrinsic and intrinsic pathways are two major routes leading to apoptosis (42). In this study, caspase 8 and 9 which are the initiator caspases in the extrinsic and intrinsic pathways, respectively were candidate for evaluation of apoptosis induction. 

The concentration of the caspase 9 in the pups livers of the treated group increased significantly but, in the same pups a significant change was not observed in the caspase 8 levels compared to control pups. Our results suggest that AgNPs may excite apoptosis in the offspring’s livers probably through the intrinsic pathway. In our previous study, after maternal exposure to AgNPs (25 mg/Kg) was observed an increase in caspase 9 levels, but not the caspase 8 levels in the brain of rat pups (18).

 In the intrinsic pathway, several members of the BCL-2 protein family (BAX, BAK, BID) increase mitochondrial permeability and then pro-apoptotic factors, such as cytochrome C release from the intermembrane space of mitochondria into the cytosol that lead to activation of initiator caspase 9 and effector caspases 3 and 7 (40, 43). Piao *et al*. (2011) evaluated the cytotoxic effects of AgNPs on the human liver derived cell lines. The mitochondria dependent apoptotic pathway was demonstrated via modulation of Bax and Bcl-2 expressions, disruption of mitochondrial membrane, cytochrome C release and activation of caspases 9 and 3 (44). 

By noncytotoxic doses of silver nanoparticles, the expression of genes associated with apoptosis and cell cycle progression was also induced in human hepatoma (45). Caspase 3 activity assay results showed that the minimum dose of AgNPs (7-20 nm) for the onset of apoptosis in primary liver cells isolated from Swiss albino mice was 12.5µg/mL (46). These studies confirmed that AgNPs could induce apoptosis in liver cell lines. However, there is a lack of data about whether maternal exposure to nanoparticles can induce apoptosis in the livers of the offspring. A few studies have demonstrated that prenatal exposure to nanoparticles induces apoptosis in the developing central nervous system (CNS). For example, Shimizu *et al*. (2009) indicated that after TiO2 exposure to pregnant mice on gestational days (GD) 6, 9, 12 and 15, expression of hundreds of genes associated with apoptosis were altered in the brain of male pups (47).Prenatal exposure to TiO2 (after maternal exposure on GD 3, 7, 10 and 14), induced apoptosis in the olfactory bulb and the mitral cells of newborn mice (48).

We suggest that potential of apoptosis induction of AgNPs on the developing liver can be similar to those in the brain. However, further studies are needed to clarify this field.

## Conclusions

Our results indicated that the reduction of GSH level and GPX activity and the addition of MDA and caspase 9 levels, as well as accompanying histological changes in the developing livers were influenced by AgNPs after maternal exposure. These alterations could be attributed to the oxidative stress induction that in turn might lead to onset of apoptosis. More research is needed for better understanding of the AgNPs toxicity on the developing liver.
